# Effects of remote ischemic conditioning on sleep complaints in Parkinson's disease–rationale, design, and protocol for a randomized controlled study

**DOI:** 10.3389/fneur.2022.932199

**Published:** 2022-07-25

**Authors:** Qiling Ji, Xuemei Wang, Wenbo Zhao, Melissa Wills, Ho Jun Yun, Yanna Tong, Lipeng Cai, Xiaokun Geng, Yuchuan Ding

**Affiliations:** ^1^Department of Neurology, Beijing Luhe Hospital, Capital Medical University, Beijing, China; ^2^Department of Neurology, Xuanwu Hospital, Capital Medical University, Beijing, China; ^3^Department of Neurosurgery, Wayne State University School of Medicine, Detroit, MI, United States

**Keywords:** remote ischemic adaptation, Parkinson's disease, excessive daytime sleepiness, non-motor symptoms, non-pharmacologic treatment

## Abstract

**Objective:**

Sleep disturbances are common non-motor symptoms of Parkinson's disease. The symptoms affect the quality of patients' life by impeding normal sleep cycles and causing excessive daytime sleepiness. Remote Ischemic Conditioning (RIC) is a therapy often used for ischemic stroke patients to minimize infarct size and maximize post-stroke neurological function. Animal experiments have shown that RIC plays a protective role for retinal ganglion cells and other critical areas of the brain of Parkinson's disease. However, whether RIC improves excessive daytime sleepiness (EDS) for patients with Parkinson's disease remains to be determined.

**Methods:**

This is a single-center, double-blind, and randomized controlled trial, which includes patients with Parkinson's disease with EDS. All recruited patients will be randomly assigned either to the RIC or the control group (i.e., sham-RIC) with 20 patients in each group. Both groups receive RIC or sham-RIC treatment once a day for 28 days within 24 h of enrollment. Epworth Sleepiness Scale (ESS), Pittsburgh Sleep Quality Index (PSQI), Parkinson Disease Sleep Scale-2 (PDSS-2), Parkinson's Disease Questionnaire39 (PDQ39) score scales, and adverse events, such as inability to tolerate the treatment leading to suspension of the study or objective signs of tissue or neurovascular injury caused by RIC and/or sham-RIC are evaluated at 7, 14, 28, and 90 days after enrollment.

**Results:**

The primary goal of this study is to assess the feasibility of the treatments in patients with Parkinson's disease by measuring serious RIC-related adverse events and any reduced incidence of adverse events during the trial and to study potential efficacy, improvement of patients' excessive daytime sleepiness, quality of life-based on ESS, PSQI, PDSS-2, and PDQ39 scores. The secondary goal is to confirm the safety of the treatments.

**Conclusion:**

This study is a prospective randomized controlled trial to determine the safety, feasibility, and potential efficacy of RIC for patients with Parkinson's disease associated with EDS.

## Introduction

Parkinson's disease is the second most common neurodegenerative disease following Alzheimer's disease, affecting more than 10 million people worldwide (1, 2). Because it has a progressive and chronic course, improving the quality of patients' life is vital. Sleep disturbances are common non-motor symptoms of Parkinson's patients, affecting ~90% of this population (3), and can significantly affect the quality of life (4–8).

Excessive daytime sleepiness (EDS) is a common sleep disorder of Parkinson's disease with an incidence rate as high as 16–50%. EDS limits patients' ability to independently execute activities of daily living (9). The mechanisms of EDS in Parkinson's disease are complex and involve different areas of neuronal degeneration of the brain. Decreased dopamine levels, blunting of circadian melatonin release, and degeneration of human photosensitive retinal ganglion cells are potential causes of sleep and circadian rhythm disorder of Parkinson's disease (3, 10, 11). Human melanopsin-containing retinal ganglion cells (mRGCs) are retinal photoreceptors regulating pupillary light reflection and diurnal light concentration. They are found in the suprachiasmatic nucleus, responsible for regulating circadian rhythm as well as mood and sleep behaviors. MRGCs are found to be damaged in Parkinson's disease and this is thought to be one of the main mechanisms of circadian rhythm disorders in Parkinson's disease (12). In other words, protecting mRGC can be beneficial for maintaining circadian rhythm and ultimately improving the quality of life of patients with Parkinson's disease (11).

Although there are treatment options considered for EDS, they are not without challenges. Examples include modafinil, methylphenidate, caffeine, istradefylline, and atomoxetine. Unfortunately, these drugs have not been carefully evaluated for EDS associated with Parkinson's disease and carry adverse effects (8, 13–16).

Remote ischemic conditioning (RIC) refers to a therapy in which a short-term sublethal blood flow block is administered to a distal limb, which induces endogenous protection with several vasoactive and neurogenic biochemicals, protecting the vital organs from destructive ischemic injury (17). Numerous studies have shown that RIC can induce anti-apoptosis, anti-inflammation, anti-oxidation, and mitochondrial regulation, and improve neural function (18–24). RIC maximizes motor and cognitive function and reduces the size of penumbral infarcts after ischemic stroke to similar extents to physical exercise therapy (25, 26). RIC also has a benign effect on sleep (27). In an animal study, RIC selectively increases the daily sleep volume of non-rapid eye movement (NREM), or the sleep state for tissue recovery, repair, and removal of metabolic wastes, and plays an important role in neuroprotection of ischemic adaptation (27). Kim has found that RIC reduces the loss of functional dopaminergic cells in the substantia nigra pars compacta (SNC) and indicates its potential therapeutic role (28). In addition, RIC has been reported to promote the survival of retinal ganglion cells for the regulation of sleep-wake cycles after various optic nerve injuries (29–32). Gidday further notes that RIC protects the retina from acute and chronic injury, supporting its role as a therapy for sleep-wake cycles (33).

This study hypothesizes that RIC reduces the loss of dopaminergic cells in the brain and improves the survival of retinal ganglion cells–including mRGCs–in Parkinson's disease, resulting in improvement of circadian rhythm disorders and reduction of EDS and sleep dysfunctions. This study conducts a single-center, double-blind, randomized controlled trial to evaluate the safety and feasibility of RIC as a non-pharmacologic therapy to reduce EDS and improve the quality of life of patients with Parkinson's disease. The findings of this study can bring more hope to the community of patients with Parkinson's disease.

## Subjects and methods

### Study design

All participants will be informed of the clinical research and the requirements for giving informed consent. The research plan and informed consent form are to be approved by the regional ethics committee; the trial has been registered at www.chictr.org.cn (Registration number: ChiCTR2200058430).

Forty patients are to be randomly assigned to the intervention (“RIC”) or control group (“sham RIC group”) with 20 patients in each group. Within the 24 h of grouping, each group starts RIC or sham RIC treatment for 45 min once a day for 28 days.

### Patient population

Participants will be recruited from outpatient and inpatient centers. The enrollment criteria are as follows: (1) diagnosis of Parkinson's disease according to MDS clinical criteria; (2) age of 18–90 year olds; (3) ESS score ≥10; (4) written informed consent obtained from the participants or legally authorized representative. Exclusion criteria are the following: (1) contraindication of ischemic conditioning (e.g., severe soft tissue injury, fracture, and peripheral vascular disease of bilateral upper extremities); (2) hemodynamic instability (i.e., systolic blood pressure > 180 mmHg, diastolic blood pressure > 110 mmHg, heart rate < 40 beats/min or > 100 beats/min, percutaneous oxygen saturation ≤ 92%, or body temperature ≥ 38.5°C); (3) life expectancy ≤ 1 year; (4) unstable comorbidities associated with the lungs, liver, and kidney (e.g., severe abnormal pulmonary function, hepatic insufficiency, renal insufficiency); (5) coagulopathy or active bleeding; (6) complications associated with acute coronary syndrome or severe arrhythmia; (7) pregnancy or nursing; (8) participation in another ongoing clinical trial.

Epworth Sleepiness Scale (ESS) is widely utilized in clinical settings to evaluate the severity of EDS. A score of 10 is typically used as a cutoff to indicate sleepiness with higher scores for more sleepiness (34). In this study, those with ESS ≥ 10 are recruited.

### Randomization

All registered patients will be randomly assigned to the intervention group or the control group with a ratio of 1:1. Randomizing the sequence column order will be performed according to the predefined table generated by a computer software program. The random sequence will be hidden in a closed opaque envelope. Before initiating the study, the envelopes will be prepared by research assistants who are not directly involved in the research project. After recording baseline measures, participants will be randomly allocated to either the RIC or control group by the treating physicians who will open the sealed opaque envelopes.

### Interventions

**Figure 1:**
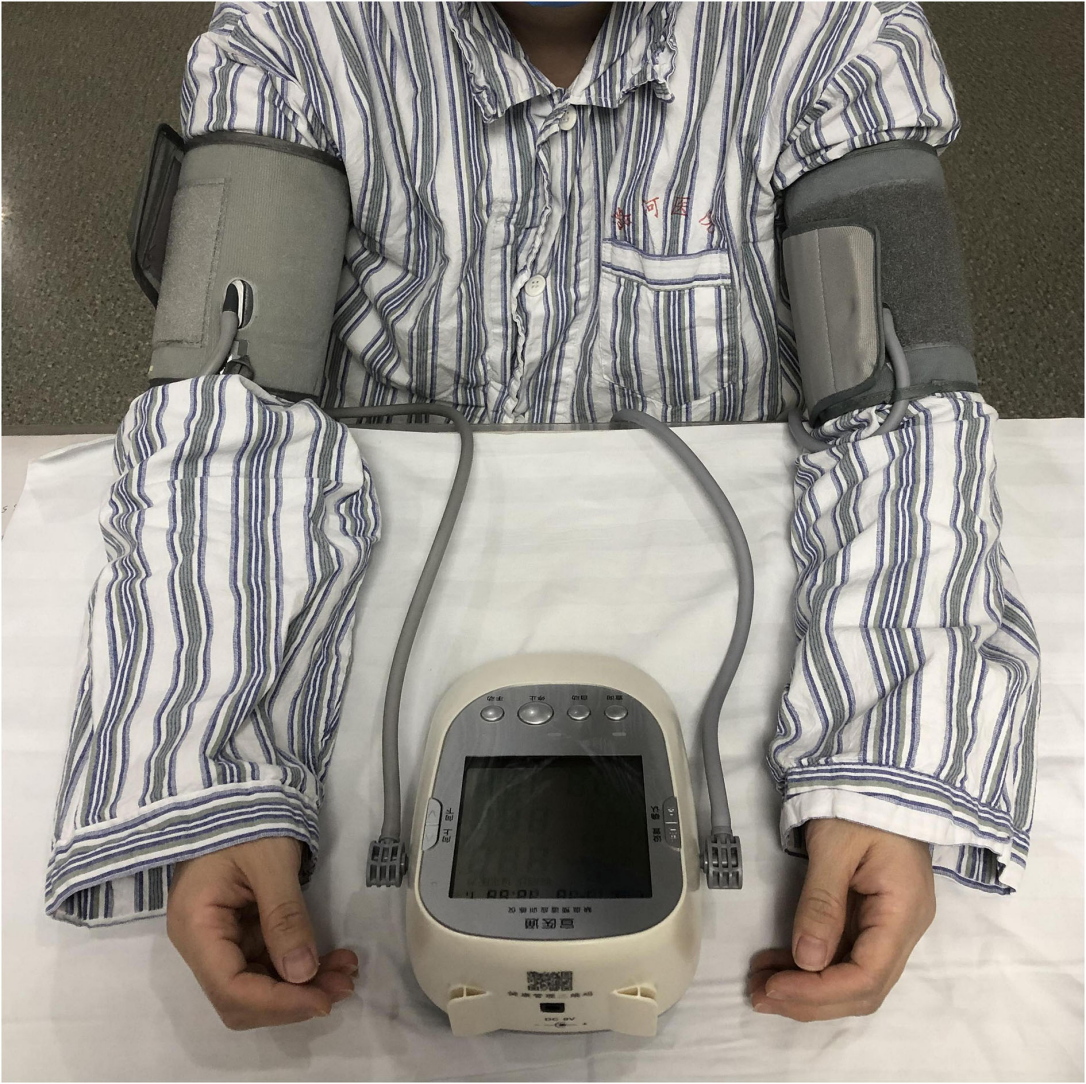
Remote ischemic conditioning (RIC) with an electronic tourniquet cuff device.

**Figure 2:**
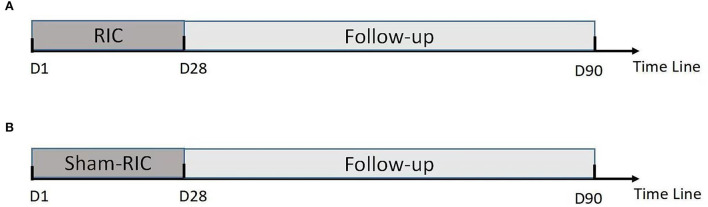
Procedure timeline **(A)** RIC group; **(B)** Control group (Sham-RIC).

Within 24 h of enrollment, an RIC operation is to be performed *via* bilateral limb ischemic preconditioning (Doctormate, IPC-906D, produced by Beijing Renqiao Institute of Neuroscience). Electronic tourniquet cuffs are placed on both arms. Participants in the intervention group undergo five cycles of cuff inflation to 200 mmHg for 5 min, followed by deflation for 5 min (Figure 1). In other studies, physical treatments, such as bright light therapy (35) and multidisciplinary intensive rehabilitation (36), have been applied to patients for 28 days to alleviate sleep disorders of Parkinson's disease. Additionally, RIC has been used for patients from 7 to 300 days (37–43). As the first step to determine safety, the duration of RIC in this study is decided to be 28 days. Patients in the sham-RIC group receive the same procedure except the maximum inflation pressure is 60 mmHg (40) (Figure 2).

Epworth Sleepiness Scale, the Pittsburgh Sleep Quality Index (PSQI), and Parkinson's Disease Sleep Scale-2 (PDSS-2) are to be used to assess the common sleep problems of Parkinson's patients (44, 45). The Parkinson's Disease Questionnaire 39 (PDQ39) is to be used to assess the quality of life of Parkinson's patients (46), which is administered by trained investigators blinded to the treatment assignment. The assessments are performed on the day of enrollment for baselines and 7, 14, 28, and 90 days after the enrollment.

Patients will continue their home medications to manage chronic conditions, including Parkinson's disease, according to the recommendations of the International Parkinson and Movement Disorder Society (15, 47).

### Outcomes

#### Outcomes for feasibility and efficacy

The primary outcomes of this study are to assess the feasibility and efficacy of RIC from changes of ESS, PSQI, PDSS-2, and PDQ39 scores from the baselines to post-treatment on days 7, 14, 28, or 90. This study will determine (1) whether ESS, PSQI, PDSS-2, and PDQ39 can be performed in the patients and (2) the differences between ESS, PSQI, PDSS-2, and PDQ39 in patients with or without RIC. Certified therapists and assistants will collect data from the subjects and the obtained information will be used for statistical analysis.

#### Outcomes for safety

The secondary outcome of this study is to assess the safety of RIC. Any serious RIC-related adverse events are the following: (1) inability to tolerate RIC or sham-RIC procedures leading to the suspension of the study; (2) signs of tissue or neurovascular injury caused by RIC and sham RIC operations (e.g., palpation of distal radial artery pulsation, visual inspection of local edema, erythema and skin damage, or palpation of tenderness). All adverse events will be independently determined by trained individuals of the research group who are blinded by random grouping.

### Sample size estimation

This is a phase 1 test measuring the safety and feasibility of RIC. Because there has not been a clinical study on RIC for Parkinson's patients, there is no data for reference at this point. Hertzog (48) suggests that 10–20 patients in each group are enough to evaluate the feasibility of a pilot study. Dobkin et al. explain that most phase 1 clinical rehabilitation pilot studies start with samples of 6–12 participants (49). Based on studies addressing the safety and feasibility of RIC, this project targets 20 patients in each group. The results of this study will be used not only to determine the safety and feasibility of RIC but also to estimate the sample size and calculate the power required for the phase 2 test in the future.

### Statistical analysis

Data are obtained from all patients who complete the study protocols and follow-ups. They will be analyzed by per-protocol (PP) analysis. Statistical analysis will be performed using SPSS version 19 (SPSS Inc., Chicago, IL, USA). *P* < 0.05 will be considered statistically significant. Demographics and clinical characteristics will be analyzed for descriptive statistics. Continuous and categorical data will be presented as mean values, standard deviations, number values, and percentages. Continuous variables consistent with the normal distribution will be compared by the independent samples *t*-test or ANOVA or rank-sum test. Categorical variables will be compared by chi-square testing.

## Discussion

Parkinson's disease is a debilitating movement disorder associated with progressive conditions, including sleep disorders, which impair patients' quality of life. While medications are available to manage these issues, patients with Parkinson's disease often are more susceptible to their adverse effects or unable to perform rehabilitative exercises. There is a need for therapies that are not only effective but also accessible to patients with Parkinson's disease.

Because of its successful application in managing myocardial infarction, RIC has been applied to other pathological processes and organ systems to minimize ischemia and reperfusion injury. RIC is an ideal therapy for those with declined mobilities and decreased motivation because it is passively administered.

In addition to the protective effect on ischemic tissue injury, studies have shown that RIC has a neuroprotective (50) and therapeutic effect on sleep (27). Animal studies have found that RIC can rescue approximately half of the functional dopaminergic neurons in the substantia nigra pars compacta damaged by Parkinson's neurotoxin, 1-methyl-4-phenyl-1,2,3,6-tetrahydropyridine (MPTP) (28, 51). RIC has been shown to promote the survival of retinal ganglion cells after various injuries, which plays a large role in the EDS of Parkinson's disease (29–33). Although many studies have proved the safety and effectiveness of RIC in preventing and treating stroke (37), RIC has not been utilized for patients with chronic neurodegenerative diseases. The protective effect of RIC on the brain and retinal optic ganglia suggests that RIC could improve EDS of Parkinson's disease. The duration of RIC in this study is set to be 28 days for safety considerations.

Excessive daytime sleepiness is a common non-motor symptom of Parkinson's disease and manifests as an inability to remain awake during the daytime, fatigue, and sudden collapse. Due to severe dyskinesia in the middle and late stages, patients with Parkinson's disease rely on others for their daily activities. Non-pharmacologic treatment options are currently limited and available therapies, including sports rehabilitation therapy, cognitive behavioral therapy, light treatment, repetitive transcranial magnetic stimulation, and deep brain stimulation (18), are not universally suitable for patients with Parkinson's disease in different stages of their illness. In contrast, RIC is inexpensive and easy to administer especially for patients with risk of falls, limb movement disorder, and inability to participate in traditional rehabilitative activities.

This project will administer RIC to patients with Parkinson's disease in different stages to determine its safety and feasibility. Effectiveness in reducing EDS will be analyzed as well. The results could provide an opportunity to develop a non-pharmacological treatment that is easy and simple to administer for EDS and benefits the community of patients with Parkinson's disease.

## Ethics statement

The studies involving human participants were reviewed and approved by Ethics Committee of Beijing Luhe Hospital Affiliated to Capital Medical University. The patients/participants provided their written informed consent to participate in this study.

## Author contributions

QJ, XW, WZ, MW, YT, and LC prepared the manuscript. QJ, XW, WZ, MW, HY, YD, and XG designed the study and revised the manuscript. All authors contributed to the article and approved the submitted version.

## Funding

This work was partially supported by the National Natural Science Foundation of China (82072549 and 81871838), the Youth Plan of Beijing Luhe Hospital, Capital Medical University (LHYY2021-LC08), and the Laboratory Development Funds of Luhe Hospital (2022).

## Conflict of interest

The authors declare that the research was conducted in the absence of any commercial or financial relationships that could be construed as a potential conflict of interest.

## Publisher's note

All claims expressed in this article are solely those of the authors and do not necessarily represent those of their affiliated organizations, or those of the publisher, the editors and the reviewers. Any product that may be evaluated in this article, or claim that may be made by its manufacturer, is not guaranteed or endorsed by the publisher.
